# Cosmopolitan cities: the frontier in the twenty-first century?

**DOI:** 10.3389/fpsyg.2015.01459

**Published:** 2015-10-14

**Authors:** A. Timur Sevincer, Shinobu Kitayama, Michael E. W. Varnum

**Affiliations:** ^1^Department of Psychology, University of HamburgHamburg, Germany; ^2^Department of Psychology, University of MichiganAnn Arbor, MI, USA; ^3^Department of Psychology, Arizona State UniversityTempe, AZ, USA

**Keywords:** independence, voluntary settlement, cultural change, cosmopolitanism, priming, goal pursuit

## Abstract

People with independent (vs. interdependent) social orientation place greater priority on personal success, autonomy, and novel experiences over maintaining ties to their communities of origin. Accordingly, an independent orientation should be linked to a motivational proclivity to move to places that offer economic opportunities, freedom, and diversity. Such places are cities that can be called “cosmopolitan.” In support of this hypothesis, Study 1 found that independently oriented young adults showed a preference to move to cosmopolitan rather than noncosmopolitan cities. Study 2 used a priming manipulation and demonstrated a causal impact of independence on residential preferences for cosmopolitan cities. Study 3 established ecological validity by showing that students who actually moved to a cosmopolitan city were more independent than those who either moved to a noncosmopolitan city or never moved. Taken together, the findings illuminate the role of cosmopolitan settlement in the contemporary cultural change toward independence and have implications for urban development and economic growth.

## Introduction

In the twenty-first century, in most Western countries (and in many other world regions) people frequently move to places that offer better opportunities, self-realization, and/or new experiences. Within the U.S. for example, about seven million people move to another state each year (U.S. Department of Commerce, 2014) and a substantial portion of them move to major city centers (Ihrke et al., 2011). Major Western city centers tend to enjoy ethnic, sexual, and intellectual diversity and stimulation that go hand in hand with weakened faith in traditional conventions, high levels of residential and relational mobility, and greater economic and social opportunities. Because they offer freedom from traditions and social conventions, as well as opportunities for those who do not fit neatly with the existing social order, cosmopolitan cities may be analogous to certain geographical frontiers (e.g., North America for many Europeans during the 16–18th centuries and the Western and Mountain West regions for East Coast residents in the US during the 18 and 19th centuries). Drawing on earlier theorizing on frontier settlement and the “spirit of independence” (Kitayama et al., 2006, 2014), the current work was designed to test the hypothesis that modern cosmopolitan cities are likely a magnet for independently oriented people.

### Cosmopolitan cities

We conceptualize cosmopolitan cities as urban areas whose cultures emphasize values including autonomy, freedom, egalitarianism, and mutual respect (Mosterin, 2005). Cosmopolitan cities tend to be centers of both scientific and economic innovation and home to global financial networks (see e.g., Global City Index, Sassen, 2001), and they are typically ethnically diverse and racially tolerant (Florida, 2002). Moreover, because of their diversity and open-mindedness, cosmopolitan cities offer opportunities for unique and novel experiences. We also view creativity and excitement as key features that define life in cosmopolitan cities (Muench, 1991). Cosmopolitan cities as defined here are likely to be mostly Western European and North American cities, such as Berlin and London in Europe, and New York and San Francisco in the U.S.

There are two general traditions of research on cosmopolitanism. In one line of work, some social scientists, especially geographers and economists, have defined cosmopolitanism primarily in terms of the degree to which a city is ethnically diverse. The concept is thus often operationalized in terms of the proportion of the inhabitants who are foreign born (e.g., Short, 2004, 2007). However, other scholars, especially those in philosophy, political science, and sociology, have offered broader definitions of cosmopolitanism, focusing on commitment to universalistic values and mutual respect among different groups (Appiah, 2006), greater levels of freedom (Mosterin, 2005), and egalitarianism (Gilroy, 2005). Our current work draws on both of these approaches, defining cosmopolitanism broadly in terms of both diversity and commitment to universalistic values. Whereas diversity fosters such values, the values themselves may facilitate diversity.

In combination with their greater economic resources, the egalitarian, free-spirited ethos of cosmopolitan cities may offer abundant opportunities for wealth and success for those with talents, new ideas, and a willingness to take chances (Kitayama et al., 2014). These opportunities are often associated with the high-tech information industry, research and development, arts, fashion, media, and the music businesses. At the same time, precisely because these opportunities are relatively independent of people's socioeconomic and ethnic backgrounds and not linked to stable communities and traditional social networks, the risk of taking on such opportunities can be substantial. Cosmopolitan cities often harbor high-risk/high-return enterprises that are open to those willing to take risks. In short, because cosmopolitan cities provide opportunities for wealth, success, freedom, egalitarianism, and diversity, and because people oriented toward independence rather than interdependence value individual success, autonomy, universalism, and uniqueness, independently (vs. interdependently) oriented people should prefer cosmopolitan cities as residential destinations.

### Independent orientation and frontier settlement

A key component of independent social orientation is a view of the self as defined by a set of internal attributes such as motives, competences, and personality traits (Markus and Kitayama, 1991; Kitayama et al., 2007). These internal attributes, in turn, are seen as guiding one's actions. Independent social orientation is typically juxtaposed against interdependent orientation. By interdependence we mean a view of the self as socially embedded. The interdependent self is defined by a set of relational attributes such as social roles, duties, and obligations. These relational attributes, in turn, are seen as guiding one's action. Compared to persons with an interdependent orientation, those with an independent orientation are more inclined to pursue personal goals, strive for freedom and autonomy, and seek uniqueness and diversity (Markus and Kitayama, 1991; Kim and Markus, 1999; Kim and Drolet, 2003; Kitayama et al., 2007).

The idea that independent persons are attracted to places that represent opportunities for success, autonomy, and self-realization is consistent with a cumulative body of studies on frontier settlement. Historically, the proposition that settlement in a frontier is linked with an independent, egalitarian mentality is known as the Turner thesis (Turner, 1920). In recent years, the voluntary settlement hypothesis (Kitayama et al., 2006) has further elaborated the link between frontier settlement and independent mentality by suggesting that because frontier regions represented opportunities for wealth and freedom, they attracted large numbers of highly independent voluntary settlers. Moreover, these independent settlers were a crucial factor in shaping the contemporary American cultural ethos of individualism and independence. In line with the voluntary settlement hypothesis, studies on regional differences in independence have shown for example that Western regions of the U.S., which once constituted the frontier, are more independent than Eastern regions as assessed by various indicators of independence, such as explicit values (Kitayama et al., 2010), state-level census-type data (e.g., divorce rate, percentage of nonconventional baby names, non-conformist voting, Vandello and Cohen, 1999; Varnum and Kitayama, 2011; Varnum, 2013), self-concept, and well-being (Plaut et al., 2002). Moreover, countries that were established by emigrants from Europe were found to be more independent than the emigrants' regions of origin. This is true for the U.S. as a whole (Kitayama et al., 2009) as well as Canada, Australia, and New Zealand (Varnum and Kitayama, 2011). Finally, the settling of frontiers has been shown to have parallel effects outside of Western cultural contexts; residents of Hokkaido, a northern island of Japan that at the turn of the twentieth century was settled by peasants and farmers from the Japanese mainland, are more independent than mainland Japanese (Kitayama et al., 2006; Yamawaki, 2012; Ishii, 2014; Ishii et al., 2014). This body of work supports the idea that independently oriented people feel attracted to lands of opportunity such as frontiers.

Although on the surface the Wild West and contemporary San Francisco appear quite different for example, in contrast to the historical frontiers, cosmopolitan cities are densely populated areas with existing infrastructure and institutions, they in fact share similarities. These include opportunities for economic success particularly in high risk/high reward enterprises, high social mobility, weakened traditional conventions, and opportunities for adventure and novel experiences. Further, the above similarities suggest that similar factors may motivate modern migrants to cosmopolitan cities as did those who migrated to historical frontiers. In sum, drawing on the general idea that places that offer opportunities for personal goal pursuit, self-realization, autonomy, and novel experiences attract people oriented toward independence (vs. interdependence), focusing on modern day migration we tested whether cosmopolitan cities attract independent settlers.

### Overview of the present work

We conducted three studies to examine the hypothesis that independently oriented people are attracted to cosmopolitan cities. Using a scenario method, we first tested whether people who chose a hypothetical move to cosmopolitan cities would be more independent that those who chose to move to noncosmopolitan cities and those who chose not to move at all (Study 1). Further, we tested whether there is a causal link between independence and preference for cosmopolitan cities by manipulating social orientation (Study 2). Finally, we investigated the ecological validity of the relationship by testing whether students who actually settled in a cosmopolitan city would be more independent compared to those who settled in a noncosmopolitan city or those who did not move (Study 3). All studies were conducted in Germany, a Western culture.

## Study 1: independence and residential preference

To test our hypothesis that independently oriented people feel attracted to move to cosmopolitan cities rather to noncosmopolitan cities or not moving at all we used a scenario method; we presented participants with the hypothetical choice of whether they would change their place of residence and if so, we asked where they would move to. One domain that is particularly relevant to voluntary settlement is goal pursuit (Oettingen et al., 2008): People who feel attracted to places that represent opportunity should show a strong orientation toward pursuing personal and independent goals (Kitayama et al., 2014). Therefore, our principal indicator of independence was orientation toward personal (vs. relational and collective) goal pursuit. In addition, because people who feel attracted to settle in such places should also be more self-reliant and autonomous, we measured subjective connectedness to other people as an additional indicator of independence. We predicted that participants who choose cosmopolitan cities as the destinations of their moves would be more independent than those who choose noncosmopolitan cities and those who choose not to change their residence.

Because people's socioeconomic status (SES) may influence their residential preferences, for example, many affluent people chose to live in cosmopolitan cities (Murray, 2012), we assessed SES. Moreover, because people may prefer moving to cities that are similar to their hometown and/or to places that are near their hometown, we also calculated the cosmopolitanism of participants' hometown and the distance between participants' preferred destination city and their hometown. This enabled assess the relationship between independence and cosmopolitan residential preferences controlling for these variables.

### Methods

#### Participants and design

One hundred and twenty-six young adults from Germany (31 men, 93 women, and two unidentified; *M*_age_ = 28.3 years) completed the study online. The study was advertised on a free access social networking website as a study on life tasks and social relationships. Participation was voluntary and participants indicated their acceptance of the consent form by clicking on a link. Participants were able to win gift cards in a lottery for their participation.

#### Pilot

To determine which German cities are perceived as cosmopolitan and which are not by lay Germans, we conducted a pilot study. Drawing on our definition of cosmopolitan cities as urban areas that are characterized by abundant economic opportunities, diversity, and moral commitments to universalistic values such as freedom, autonomy, tolerance, and open-mindedness, we adopted eight items from a scale assessing perceived cosmopolitanism by Sevincer et al. (submitted). Sevincer and colleagues verified the validity of the scale by correlating it with objective indicators of cities' cosmopolitanism (e.g., employment rate, proportion of people from ethnic minorities, proportion of people with creative professions). We used the following items:

1. “is a cosmopolitan city,”
2. “is a multicultural city,”
3. “provides opportunities to build one's career,”
4. “is an exciting city,”
5. “is tolerant toward minority groups,”
6. “has an active art scene,”
7. “is a center for science and research.”
8. “is a provincial city” (reverse coded).

Specifically, in an online survey we presented 22 German students (five men, 16 women, one unidentified; *M*_age_ = 20.73) with the names of 30 biggest German cities in random order. Students were asked to indicate for each city how much each of the eight statements applies to that city on a 7-point scale ranging from 1 (*not at all*) to 7 (*very much*).

To explore whether the scale indeed measures one single construct, perceived cosmopolitanism, we explored the factor-structure of the scale. Specifically, we conducted principal-components analysis (PCA) with varimax rotation on all eight items across the 30 cities. The Kaiser-Meyer-Olkin measure verified sampling adequacy, KMO = 0.91 (“superb,” Hutcheson and Sofroniou, 1999; Field, 2009) and Bartlett's test of sphericity, $\chi_{\left(28\right)}^{\text{2}}=3425.65$, *p* < 0.001, indicated that correlations among the items were sufficiently large. The PCA extracted only one component that accounted for 63% of the variance (Eigenvalue: 5.05). The Eigenvalues of all other components were smaller than 0.82 and the scree-plot showed an inflection point at Component 2. This pattern indicates that our scale is unidimensional.

The unidimensional nature of our scale implies that the eight items were highly correlated suggesting that the scale is internally consistent. To further investigate the internal consistency of the scale however, we calculated alpha reliabilities within as well as across cities.

To explore internal consistency *within* cities, for each of the 30 cities we calculated Cronbachs' alphas for the eight items across students. Reliabilities ranged between 0.42 and 0.91. The average α was 0.82 (*SD* = 0.09), which indicates good average reliability (Kline, 2000). This analysis suggests that students varied in how cosmopolitan they perceived one particular city to be and our scale reliably captures this variance. To explore internal consistency *across* cities, we calculated Cronbach's alpha for the mean ratings of each of the nine items across the 30 cities. Reliability was 0.91, which is excellent (Kline, 2000). This analysis suggests that the cities varied in how cosmopolitan they rated on average and that our scale reliably captures this variance.

Finally, to obtain a ranking of how cosmopolitan the cities are, we combined the eight items for each city into an overall cosmopolitan index. See Table 1 for the mean cosmopolitan index and standard deviation for each city. The cosmopolitanism of the 30 cities correlated positively with their size, *r* = 0.80, *p* < 0.001, indicating that more cosmopolitan cities were also larger cities.

**Table 1:** **Study 1: city ranking by cosmopolitanism as determined in the pilot study**.

**Rank**	**Cities**	***M***	***SD***
1	Berlin	6.52	0.33
2	Hamburg	5.89	0.81
3	Cologne	5.68	0.64
4	Frankfurt	5.50	0.79
5	Munich	5.48	1.01
6	Stuttgart	4.85	0.74
7	Düsseldorf	4.67	0.76
8	Dresden	4.63	1.12
9	Bremen	4.60	0.83
10	Hannover	4.37	0.99
11	Dortmund	4.34	0.86
12	Leipzig	4.32	0.80
13	Bonn	4.28	0.65
14	Mannheim	4.26	0.93
15	Karlsruhe	4.22	0.89
16	Münster	4.20	0.70
17	Essen	3.97	0.75
18	Bochum	3.93	0.84
19	Nürnberg	3.91	0.75
20	Augsburg	3.84	0.86
21	Aachen	3.78	0.83
22	Wiesbaden	3.57	0.95
23	Kiel	3.54	0.87
24	Duisburg	3.47	0.87
25	Wuppertal	3.45	0.87
26	Bielefeld	3.38	0.98
27	Braunschweig	3.25	0.86
28	Chemnitz	3.07	0.88
29	Mönchengladbach	3.06	0.92
30	Gelsenkirchen	3.05	0.79

#### Materials and procedure

##### Residential preferences

In the main study, to assess residential preferences, we used the following scenario. First, we asked participants to name the city or town they are currently living in. We then asked: “Would you rather stay in your current town or city or move to a different place in Germany if you had equal possibilities to study or work in both places?” If participants indicated that they would rather move to a different place we asked them to specify which German town or city they would most prefer. Eighty participants (64%) indicated that they would stay in their current town or city and 46 (36%) that they would move to a different place. Of the 46 participants who indicated their intention to move, nine listed Hamburg as their preferred city. Other cities listed included Berlin (5), Munich, (4), and Cologne (3).

##### Measures of independence

We assessed independence by two measures: Tendency toward personal goal pursuit and subjective connectedness to others.

##### Personal goal pursuit

First, to assess their tendency toward personal goal pursuit, we asked participants to list 10 life tasks they plan to carry out over the next 5 years. Two independent raters unaware of participants' cities of origin coded the listed life tasks into one of the following three categories: (a) personal; tasks that are related to personal accomplishment and success (e.g., “get a degree,” “learn to play an instrument”), (b) relational; tasks that are related to establishing or maintaining interpersonal relationships (e.g., “get to know some people,” “keep up contact to my family”), and (c) collective; tasks that are related to social identities or promoting group welfare (e.g., “join a chess club,” “support community welfare association”). Interrater agreement was 95% (κ = 0.90). The relative number of personal (vs. relational and collective) tasks was our first indicator of independence. Because we asked participants to list exactly 10 tasks, the number of personal tasks and the number of relational and collective tasks combined were inversely related; so we analyzed the number of personal tasks only.

##### Subjective connectedness

Second, to assess perceived connectedness to others we included the Inclusion of Others in the Self scale (IOSS; Aron et al., 1992). This one-item scale contained a series of pictures with two overlapping circles. One of the two circles in each picture represented the self and the other represented a specific other person or group. Participants were asked to indicate which picture best describes their relationship. The answer scales ranged from 1 (*completely separate circles*) to 7 (*almost completely overlapping circles*). We used four versions of the IOS that assessed participants' connectedness to (a) their close family members, (b) their close friends, (c) the people in their local community, and (d) other people in general. To obtain a single index of how connected participants felt to others we combined the four scales (α = 0.57) and reverse coded the combined index so that higher numbers indicate a more independent orientation.

##### Socioeconomic status

Participants completed a demographic questionnaire that included items to assess their SES. SES is typically measured as a combination of income, education, and occupation (American Psychological Association, n.d.)^1^. We thus asked participants (a) to estimate their family income on a 10-point scale ranging from 1 (*less than* 20.000 €) to 10 (*more than* 200.000 €), (b) to indicate their mother's and father's education on 6-point scales ranging from 1 (*some high school*) to 6 (*post-graduate degree*), and (c) to name their mother's and father's occupation. To measure occupational prestige two independent raters coded the named occupations for their prestige on the basis of the Hauser-Warren Socioeconomic Index (Hauser and Warren, 1997) using a 4-point scale from 1 (*no at all prestigious*) to 4 (*very prestigious*; interrater reliability: α = 0.93). To obtain a single SES indicator we first averaged mother and father education (*r* = 0.68, *p* < 0.001) to one index of parent education. Second, we averaged the coded mother and father occupational prestige (*r* = 0.32, *p* = 0.001) to one index of parent occupational prestige. We then z-transformed and averaged our three indices (family income, parent education, parent occupational prestige) into one indicator of SES (α = 0.54). On the last page of the online questionnaire participants were thanked and fully debriefed about the design and hypotheses of the study. They were also given the option to provide comments and encouraged to contact us if they had any questions.

### Results

We predicted that participants who choose cosmopolitan cities as the destinations of their moves would be more independent than those who choose noncosmopolitan cities and those who choose not to change their place of residence. To test this prediction we classified the 15 cities that participants in the pretest rated as most cosmopolitan as cosmopolitan cities, and the 15 cities that they rated as least cosmopolitan as noncosmopolitan cities.

#### Measures of independence

##### Personal goal pursuit

Table 2 presents the relative number of personal, relational, and collective tasks in each group. The number of personal tasks participants named differed between groups, *F*_(2, 123)_ = 4.12, *p* = 0.01. A planned contrast (–2, 1, 1) revealed that, as predicted, participants who preferred moving to a cosmopolitan city on average pursued more personal tasks (*M* = 7.29, *SD* = 2.03) than those who preferred moving to a noncosmopolitan city (*M* = 5.67, *SD* = 2.30) and those who preferred not to change their current place of residence (*M* = 6.51, *SD* = 1.62), *t*_(123)_ = 2.86, *p* = 0.005, *d* = 0.48. The latter two groups did not differ from each other, *t*_(96)_ = 1.48, *p* = 0.15. The observed pattern was robust, it remained significant when we classified cosmopolitan cities as only the 10 cities that were rated most cosmopolitan in the pretest, *t*_(123)_ = 2.64, *p* = 0.009, and it remained significant when we classified cosmopolitan cities as only the 5 cities that were rated most cosmopolitan *t*_(123)_ = 2.80, *p* = 0.006^2^.

**Table 2:** **Study 1: Percentage of life tasks listed by each group**.

**Task type**	**Total**	**Group**		
		**Move to cosmopolitan city**	**Move to noncosmopolitan city**	**Not move**
Personal	65.6	72.9	56.7	65.1
Relational	29.8	24.3	35.6	30.4
Collective	0.5	2.9	7.8	4.5

Moreover, because cosmopolitanism ratings were a continuous variable, we analyzed the data in yet another way. Specifically, among participants who indicated that they would move to a different place we examined the correlation between the number of personal tasks they generated and the cosmopolitanism of their preferred destination city. If the preferred destination city was not among the 30 biggest German cities from the pretest, we assigned that city the lowest value from the pretest (3.05). The number of personal tasks correlated positively with cosmopolitanism, *r* = 0.34, *p* = 0.02, indicating among participants who would like to change their place of residence, the more independent goals they pursued the more they preferred moving to cosmopolitan cities.

##### Subjective connectedness

Reversed IOS scores did not differ between groups, *F*_(2, 123)_ = 1.12, *p* = 0.33. A planned contrast (–2, 1, 1) revealed that although the means were in the predicted direction, the difference between those who preferred moving to a cosmopolitan city (*M* = 4.24, *SD* = 0.67) and those who preferred moving to a noncosmopolitan city (*M* = 3.99, *SD* = 1.00) as well as those who preferred not to change their current place of residence (*M* = 3.94, *SD* = 0.90) failed to reach statistical significance, *t*_(51.10)_ = 1.50, *p* = 0.14.

#### Control variables

##### Socioeconomic status

To investigate whether the pattern that participants who preferred cosmopolitan cities pursued more independent goals remained robust when controlling for differences in SES, we first dummy-coded participants who preferred non-cosmopolitan cities and those who preferred not to move into one group. We then estimated a GLM with the number of personal life tasks as dependent variable, group (participants who preferred cosmopolitan cities vs. all other participants) as factor, and SES as covariate. The difference in the number of personal tasks between participants who preferred cosmopolitan cities and the other participants remained significant, *F*_(1, 123)_ = 5.08, *p* = 0.03.

##### Cosmopolitanism of hometown

We assessed the cosmopolitanism of participants' hometown in the same way as we determined the cosmopolitanism of their favorite city. Specifically, we assigned participants' hometown the cosmopolitanism rating from the pretest. If a hometown was not among the cities from the pretest, we assigned that hometown the lowest value (3.05).

The cosmopolitanism of participants' home town did not correlate with the cosmopolitanism of participants' favorite city, *r* = −0.06, *p* = 0.68, indicating that participants did not simply prefer cities that are similar to their hometowns. To test whether the results remain robust when controlling for hometown cosmopolitanism, we estimated a GLM with the number of personal life tasks as dependent variable, group (participants who preferred cosmopolitan cities vs. all other participants) as factor, and hometown cosmopolitanism as covariate. The difference in the number of personal tasks between participants who preferred cosmopolitan cities and the other participants remained significant, *F*_(1, 123)_ = 7.37, *p* = 0.008.

##### Distance destination city—hometown

The distance between participants' preferred destination city and their hometown correlated positively with the number of personal (vs. relational and collective) life task they listed, *r* = 0.36, *p* = 0.01. This pattern indicates that the more independent people are the farther away they prefer to move. Moreover, the difference in the number of personal tasks between participants who preferred cosmopolitan cities and the other participants remained significant when controlling for the distance between participants' destination city and their hometown, *F*_(1, 43)_ = 7.41, *p* = 0.009.

##### City size

The size of participants' preferred cities (i.e., their destination cities if they preferred to move and their hometowns if they preferred not to change their location) did not correlate with the number of personal life tasks, *r* = 0.14, *p* = 0.12, indicating that more independent participants did not simply prefer larger cities. Because however, as reported above, more cosmopolitan cities are also larger cities, we tested whether our results remain robust when controlling for the size of participants' preferred city. The difference in the number of personal tasks between participants who preferred cosmopolitan cities and the other participants remained marginally significant when controlling for city size, *F*_(1, 123)_ = 3.64, *p* = 0.059.

### Discussion

Young adults who showed interest in moving to a cosmopolitan city were more independent (as indicated by greater preference for personal goal pursuit) than either those who were interested in moving to a noncosmopolitan city, or those who were not interested in moving. This effect was robust, remaining significant when controlling for participants' SES, the cosmopolitanism of their hometown, the distance between their preferred city and their hometown, and the size of their preferred city. We observed a similar pattern for the relationship between another indicator of independence (subjective connectedness to others) and residential preferences, however the effect did not reach statistical significance. Overall these results were consistent with our hypothesis that independent people are attracted to cosmopolitan cities. However, Study 1 used a correlational design. Therefore, to draw causal inferences, we conducted Study 2, which used an experimental design.

## Study 2: causal effect of independence on residential preferences

To explore whether an independent orientation causes people to feel attracted to cosmopolitan (vs. noncosmopolitan) cities, we tested whether priming participants with independence (vs. interdependence) would enhance their preference for cosmopolitan (vs. noncosmopolitan) cities and their self-reported willingness to move.

### Methods

#### Participants and design

Seventy psychology students from the University of Hamburg (17 men, 52 women, one unidentified; *M*_age_ = 23.8 years) took part in the study. The study was conducted online and advertised on the Psychology Department website as a study on language processing and residential preferences. Students indicated their acceptance of the consent form by clicking on a link. They received course credit. We randomly assigned students to one of the two priming conditions (independence vs. interdependence).

#### Priming manipulation

We used a scrambled sentence task developed by Kuehnen and Hannover (2000). On the first page of the online questionnaire students were presented with 22 items. Each item consisted of five words. Students' task was to create a correct sentence with four out of the five words. In the independence priming condition the words could be arranged to form sentences that implied an independent mindset. For example, “unique like being dissociate I” could be arranged to form “I like being unique.” In the interdependent condition the words could be arranged to form a sentence that implied an interdependent mindset. For example, “I together team my support” could be arranged to form “I support my team.” Because this priming manipulation has been successfully used in numerous previous studies to induce an independent (vs. interdependent) mindset (e.g., Kuehnen and Hannover, 2000; van Baaren et al., 2003; Hogeveen and Obhi, 2011), we did not use a manipulation check.

#### Dependent variables

##### Favorite cities

Students listed three German cities in which they would most like to live if they moved out of their current city. Two independent raters from Germany coded the listed cities for cosmopolitanism. We used a 4-point coding scale ranging from 1 (*not at all cosmopolitan*; e.g., Minden) to 4 (*very cosmopolitan*; e.g., Berlin). Interrater agreement was α = 0.98. To obtain an index of how much students desired to live in cosmopolitan cities, for each student we calculated the average cosmopolitanism rating of the three listed cities.

##### City preference

Students were also presented with nine German cities. Three of the nine cities were among the five most cosmopolitan cities in the pilot study (Berlin, Cologne, Frankfurt), additional three were among the least cosmopolitan cities (Wuppertal, Bielefeld, Chemnitz), and the remaining three were somewhere in between (Dresden, Leipzig, Nürnberg). For each city, students indicated how much they would like to live there if they moved out of their current city. Answer scales ranged from 1 (*not at all*) to 7 (*very much*). To obtain an index of how much students would like to live in cosmopolitan cities relative to noncosmopolitan cities, we subtracted students' average preference for living in the three least cosmopolitan cities (α = 0.69) from their average preference for living in the three most cosmopolitan cities (α = 0.65). The three cities in between were filler items.

##### Willingness to settle in a new city

In addition, to test whether people with an independent (vs. interdependent) mentality are generally more likely to change their place of residence (Kitayama et al., 2006; Oishi and Kisling, 2009), we measured students' willingness to relocate. Specifically, we asked: “In general, how willing are you to move to another town or city?,” “How willing are you to move out of the town or city you are currently living in?,” and “How bad would it be for you if you had to move out of the town or city you are currently living in?” (reverse coded). Scales ranged from 1 (*not at all*) to 7 (*very*). We collapsed the three items into one index (α = 0.85). On the last page of the online questionnaire students were thanked and fully debriefed about the design and hypotheses of the study. They were also given the option to provide comments and encouraged to contact us if they had any questions.

### Results

#### Favorite cities

**Figure 1:**
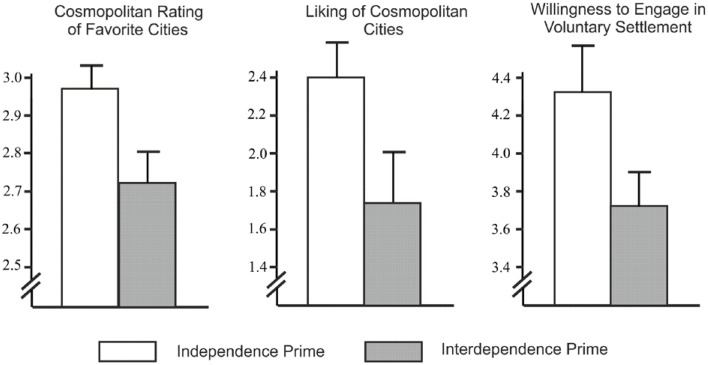
**Study 2: Average cosmopolitanism rating (±*SE*) of participants' three favorite cities (left chart), liking for cosmopolitan relative to noncosmopolitan cities (middle chart), and willingness to engage in voluntary settlement (right chart) in different priming conditions (independence vs. interdependence)**.

As predicted, the cosmopolitanism of students' three most preferred cities was higher in the independence condition (*M* = 2.97, *SD* = 0.41), than in the interdependence condition (*M* = 2.73, *SD* = 0.45), *t*_(68)_ = 2.37, *p* = 0.02, *d* = 0.55; Figure 1, left chart).

#### City preference

Also as predicted, the relative preference for cosmopolitan (vs. noncosmopolitan) cities was higher in the independence condition (*M* = 2.40, *SD* = 1.42), than in the interdependence condition (*M* = 1.64, *SD* = 1.34), *t*_(68)_ = 2.23, *p* = 0.03, *d* = 0.55 (Figure 1, middle chart).

#### Willingness to settle in a new city

Finally, reported willingness to relocate tended to be higher in the independent condition (*M* = 4.26, *SD* = 1.63), than in the interdependent condition (*M* = 3.65, *SD* = 1.19), *t*_(67.37)_ = 1.80, *p* = 0.08, *d* = 0.42 (Figure 1, right chart).

### Discussion

Study 2 extended the correlational evidence provided by Study 1 by showing that priming students with independence (vs. interdependence) resulted in a greater preference for cosmopolitan (vs. noncosmopolitan) cities, and a greater willingness to change their place of residence. This evidence supports our claim that an independent (vs. interdependent) mentality causes a greater willingness to migrate to cosmopolitan cities. Study 2 also demonstrated that people's residential preferences are influenced by psychological factors that are relatively malleable (i.e., salience of independent vs. interdependent mindsets) rather than being solely motivated by relatively stable economic and sociological factors (e.g., SES, the number and quality of available jobs at a location; Harris and Todaro, 1970). Studies 1 and 2 established that an independent orientation leads to a greater preference for cosmopolitan cities. However, so far we have only examined people's self-reported preferences rather than their actual residential decisions. Thus, to test the ecological validity of our hypothesis that independence is linked to actual migration toward cosmopolitan cities, we conducted Study 3.

## Study 3: independence and actual residential movement

The aim of Study 3 was to examine whether independence is linked to actual migration to cosmopolitan cities. To test this hypothesis we compared students in a cosmopolitan vs. a noncosmopolitan city who either voluntarily moved to the city they inhabit or were born in that city. If highly independent people are particularly motivated to settle in cosmopolitan cities, then the settlers in such cities should be more independent than those who settled in noncosmopolitan cities and those who never changed their place of residence (i.e., those residing in the city of their birth). To test our prediction, we tested matched samples from Hamburg and Braunschweig. Hamburg was among the five most cosmopolitan cities in the pilot study reported in Study 1; Braunschweig was among the five least cosmopolitan cities. Both cities are located in the Northwestern part of Germany, have a majority of protestant inhabitants, and are university towns. The universities of Hamburg and Braunschweig are both long-established universities in Germany and both rank in the middle of an international ranking on universities' reputation in research and teaching (QS World University Rankings, n. d.)^3^. We hypothesized that students who voluntarily moved to Hamburg would be more independent than those who voluntarily moved to Braunschweig and those native to Hamburg or Braunschweig.

Because in Study 1 participants who preferred cosmopolitan (vs. noncosmopolitan) cities evinced a stronger tendency toward personal goal pursuit, we took students' tendency toward personal goal pursuit as our principal indicator of independence. Moreover, we measured students' preference for uniqueness (vs. conformity) as a second indicator of independence.

As in Study 1, to control for differences in SES, we assessed students' SES. In addition, because choice can increase independence (Savani et al., 2010) it could be that cosmopolitan settlers are more independent than natives because the settlers made the choice to move to a different city, whereas the native residents of these cities simply retained their status quo without deliberately deciding whether to change their place of residence or not. We tested the validity of this alternative explanation by asking students how much choice they had in their decision to move to or remain in the city they currently resided in.

### Methods

#### Participants and design

Two hundred and seven psychology and educational sciences students (155 women and 52 men; *M*_age_ = 22.4 years) from the Universities of Hamburg (*n* = 116) and Braunschweig (*n* = 91) took part in the study. Students from Hamburg and Braunschweig did not differ in age, *t*_(118.95)_ = 1.03, *p* = 0.31. Students were tested in groups. They were told that the study was about life tasks and cognitive style. Participation was voluntary and participants signed a consent form. Each of them was given a paper and pencil questionnaire containing the experimental tasks. Students could win gift cards in a lottery. The study used a quasi-experimental design.

#### Measures of independence

##### Personal goal pursuit

First, as in Study 1, we assessed students' tendency toward personal goal pursuit by asking them to list 10 life tasks. Interrater agreement for the coding of the tasks was 98% (κ = 0.96). Table 3 presents the relative number of personal, relational, and collective tasks in each group.

**Table 3:** **Study 3: Percentage of life tasks listed by each group**.

**Task type**	**Total**	**Group**			
		**Hamburg settlers**	**Hamburg natives**	**Braunschweig settlers**	**Braunschweig natives**
Personal	69.7	75.3	67.1	66.9	67.7
Relational	26.3	23.3	29.6	26.5	26.0
Collective	4.0	1.3	3.3	6.9	6.3

##### Preference for uniqueness

Second, we assessed students' preference for uniqueness (vs. conformity) using a questionnaire by Kim and Markus (1999). The questionnaire contained 30 abstract figures composed of nine subfigures. The nine subfigures were composed of a majority of identical subfigures (common subfigures) with 1, 2, 3, or 4 subfigures that differed from the rest in terms of shape, direction, or position (uncommon subfigures). Students ranked each of the nine subfigures within each figure in the order of their preference by numbering them from 1 (*favorite*) to 9 (*least favorite*). Characteristics of subfigures (e.g., shape, direction, and position) were counterbalanced in two forms to ensure that students' preferences were due to their preference for uniqueness or conformity, not their preferences for any other particular characteristics of the subfigures. To obtain an indicator of students' propensity toward uniqueness, we calculated a preference score for the uncommon subfigures by averaging the numbers written on each of the uncommon subfigures of the 30 figures. We then reversed the preference for uniqueness score so that higher numbers indicate a more independent orientation.

#### Classification into settlers and natives

To identify whether students moved to or were native to Hamburg or Braunschweig, respectively, we asked them to list all places where they had lived for at least 1 year and to indicate for each place the year when they had moved there. Because most German students live at their parents' home until they complete high school at the age of 19 (Bien, 1996), we classified students who moved to Hamburg or Braunschweig when they were 19 years or older as settlers and those who lived there all their life or moved there before the age of 19 as natives. In Hamburg, 60 students were settlers and 56 natives; in Braunschweig 29 were settlers and 62 natives. Of the 87 settlers five (6%) were from a city that was among the five most cosmopolitan German cities in the pilot survey reported in Study 1. The pattern of results did not change if these students were omitted from the analyses. Settlers and natives did not differ in age, *t*_(205)_ = 0.53, *p* = 0.59.

#### Socioeconomic status

We used the same demographic questions as in Study 1^4^. Reliability for coding occupational prestige was α = 0.71 and for combining family income, education, and occupational prestige into one SES indicator it was α = 0.68.

#### Choice in selecting place of residence

In addition, to explore whether students voluntarily had chosen their university town, we asked: “How much personal choice did you have when you enrolled in this university?” on a 9-point scale ranging from 1 (*very little choice*) to 9 (*a lot of choice*), and “When you enrolled in this university, how much did you want to be here, or how much did you feel that you had no other options?” on a 9-point scale ranging from 1 (*no other options/forced*) to 9 (*really wanted to be here*). We combined the two items (α = 0.64). To conclude, students were thanked and fully debriefed about the design and hypotheses of the study.

### Results

#### Measures of independence

##### Personal goal pursuit

A One-Way ANOVA indicated that the number of personal life task students named differed between groups, *F*_(3, 203)_ = 5.01, *p* = 0.002. A planned contrast (–3, 1, 1, 1) revealed that, as predicted, Hamburg settlers (*M* = 7.53, *SD* = 1.27) listed more personal tasks than Hamburg natives (*M* = 6.71, *SD* = 1.32), Braunschweig settlers (*M* = 6.69, *SD* = 1.31), and Braunschweig natives (*M* = 6.77, *SD* = 1.47), *t*_(203)_ = 3.84, *p* < 0.001, *d* = 0.54. The latter three groups did not differ from each other, *t*s < 0.28, *p*s > 0.78.

##### Preference for uniqueness

Students' preference for uniqueness score did not differ between groups, *F*_(3, 203)_ = 2.08, *p* = 0.11. However, a planned contrast (–3, 1, 1, 1) revealed that, as predicted, Hamburg settlers (*M* = 5.24, *SD* = 0.81) liked the unique and plural minority subfigures more than did Hamburg natives (*M* = 4.83, *SD* = 1.01), Braunschweig settlers (*M* = 5.04, *SD* = 1.06), and Braunschweig natives (*M* = 4.83, *SD* = 1.25), *t*_(139.52)_ = 2.38, *p* = 0.02, *d* = 0.33. The latter three groups did not differ from each other, *t*s < 0.92, *p*s > 0.35.

#### Control variables

##### Socioeconomic status

Students from Hamburg had a higher SES (*M* = 0.09, *SD* = 0.80) than students from Braunschweig (*M* = −0.24; *SD* = 0.91), *t*_(177.99)_ = 2.72, *p* = 0.007. To investigate whether the observed pattern remained robust when we controlled for SES, we first dummy-coded Braunschweig settlers, Hamburg natives, and Braunschweig natives into one group. We then estimated a GLM with number of personal life task as dependent variable, group (Hamburg settlers vs. the other three groups combined) as factor, and SES as covariate. The difference between Hamburg settlers and the other three groups remained significant, *F*_(1, 202)_ = 12.86, *p* < 0.001. Analogous analyses with preference for uniqueness as dependent variable revealed that the difference between Hamburg settlers and the other three groups remained significant, *F*_(1, 202)_ = 4.98, *p* = 0.03.

##### Choice in selecting place of residence

Students were asked how much choice they had in attending their university. The mean choice rating was well above the midpoint of the 9-point scale (*M* = 6.94, *SD* = 2.07) and did not differ between the four groups *F*_(3, 203)_ = 0.66, *p* = 0.58. When we estimated a GLM with number of personal life task as dependent variable, group (Hamburg settlers vs. the other three groups combined) as factor, and the mean choice rating as covariate the difference in independence between Hamburg settlers and the other three groups remained significant, *F*_(1, 204)_ = 14.98, *p* < 0.001. For preference for uniqueness the difference also remained significant, *F*_(1, 204)_ = 5.21, *p* = 0.03. Hence, it is not likely that settlers in Hamburg are more independent because they perceive themselves to have had more of a choice in their place of residence than the other three groups.

### Discussion

Students who voluntarily moved to the cosmopolitan city of Hamburg held more personal (vs. relational and collective) goals and had a stronger preference for uniqueness (vs. conformity) than those who moved to the noncosmopolitan city of Braunschweig and those who did not change their place of residence (those native to Hamburg or Braunschweig, respectively). These effects were robust, remaining significant when controlling for differences in participants' SES. Of importance, the residential move was highly voluntary for the settlers in Hamburg and Braunschweig, respectively, and, moreover, the native residents of both cities chose to remain in their city on an equally voluntary basis. These findings argue against the alternative explanation that the settlers in Hamburg were more independent than the native residents because the settlers but not the natives chose where to live. Study 3 thus suggests that the relationships between independence and migration to cosmopolitan cities observed in Studies 1 and 2 are ecologically valid by showing that more independent people actually moved to such cities.

## General discussion

We argued that independently oriented people prefer moving to cosmopolitan cities rather than moving to noncosmopolitan cities or staying in their hometowns. Study 1 supported this pattern using a scenario method to assess residential preferences. Study 2 established a causal link between independence and preference for cosmopolitan (vs. noncosmopolitan) cities as well as willingness to generally change one's place of residence. Finally, Study 3 supported the ecological validity of our findings by suggesting that people who actually moved to a cosmopolitan city are more independent than those who moved to a noncosmopolitan city or stayed in their hometown. Moreover, we ruled out alternative explanations including differences in SES (Studies 1 and 3), the cosmopolitanism of one's hometown (Study 1), the distance between hometown and preferred destination (Study 1), and the extent to which participants had a choice in selecting their destination city (Study 3). In sum, we explored the link between independence and preference for cosmopolitan cities with different populations (students and a broader sample of internet users), using different research designs (correlational, quasi-experimental, and experimental), different research methods (scenario, survey, paper and pencil tasks, and priming), and measures that tapped various dimensions of independence (personal goal pursuit, subjective connectedness, and preference for uniqueness).

### Cosmopolitan settlement and cultural change

Over the last decades, in nearly all countries and cultures in the world a constant cultural change has been observed in the direction of independence and individualism (Inglehart and Baker, 2000; Greenfield, 2009; Grossmann and Varnum, 2015). The demographic and psychological dynamic that is associated with cosmopolitan settlement may be an important force underlying this process. Cosmopolitan city centers exist in most developed countries all over the world (e.g., London, Paris, Sydney, New York) and research suggests that our finding that independent people prefer cosmopolitan cities generalizes to other Western countries (i.e., the U.S.; Sevincer et al., submitted). However, future research should investigate whether cosmopolitan metropolises in non-Western regions, which overall tend to be more collectivist (e.g., Istanbul, Hong Kong, Singapore), might also attract independently inclined people.

We suspect that cosmopolitan settlement is a major factor in cultural shifts toward independence, because it integrates several factors known to foster independence (Kitayama and Uskul, 2011). First, commercialization and industrialization foster independence, because they require independent decisions and judgments as well as interaction with people outside of one's immediate community (Inglehart and Baker, 2000; Greenfield et al., 2003; Henrich et al., 2010). Second, migration to such cities may enhance upward economic mobility and relatively large portions of the residents of such cities are of higher SES. High SES is associated with independence (Kohn and Schooler, 1983; Lachman and Weaver, 1998; Snibbe and Markus, 2005; Grossmann and Varnum, 2011), and overall increases in SES have been linked to increased independence at the cultural level (Grossmann and Varnum, 2015). Third, high residential mobility facilitates a more independent orientation because compared to people who live in one place for an extended period, people who often change their residence are more likely to ground their identities on relatively stable internal attributes (personality traits, abilities, and skills) rather than on social roles (Oishi and Kisling, 2009). Because cosmopolitan cities are commercial and industrial centers with a sizable upper class and middle class that attract immigrants from all over the world, cosmopolitan settlement may be particularly important in shaping individuals' and cultures' orientation toward independence.

### Cosmopolitan settlement and economic growth

Economists and historians have proposed that the historical frontier was a major factor in promoting the economic development of the U.S. precisely because it attracted highly independent settlers motivated toward personal success and willing to take chances (Turner, 1920; Garcia-Jimeno and Robinson, 2009). Similarly, cosmopolitan cities today may be a crucial factor in promoting modern Western societies' economic development. Economists agree in that people are the motor force behind regional growth (Rauch, 1993; Glaeser, 2000). A regions' economic development therefore strongly depends on the region's ability to attract ambitious, productive, and creative workers (Jacobs, 1984; Trager, 2005). This ability is determined by the employment opportunities a region offers (i.e., the quantity and quality of available jobs) and by features of the regions' social environment. It has been proposed that features such as diversity, tolerance, authenticity, and openness to new ideas play a major role in attracting highly ambitious and creative people (Florida, 2002). Our research supports this notion by showing that cosmopolitan cities attract people with strong motivations toward personal goal pursuit and individual accomplishment. In terms of political implications for urban development, our findings thus suggest investing in features that make a city more cosmopolitan (e.g., culture, music, media, equal treatment, international infrastructure, and science) should spur economic growth.

### Processes involved in cosmopolitan settlement

Our research also sheds light on the processes by which cosmopolitan settlement fosters independence. According to the voluntary settlement hypothesis (Kitayama et al., 2006), two important processes are involved in voluntary settlement: First, independently oriented people are likely to *self-select* for settlement. Second, engaging in voluntary settlement *reinforces* peoples' orientation toward independence. Studies 1 and 2 suggest that *self-selection* is involved in cosmopolitan settlement. In Study 1 more (vs. less) independent participants reported a greater readiness to settle in cosmopolitan cities and in Study 2 participants primed with independence (vs. interdependence) were more willing to relocate to a cosmopolitan (vs. noncosmopolitan) city.

To investigate whether the process of *reinforcement* of independence during settlement is also involved, we explored whether, in Study 3, the settlers in the cosmopolitan city of Hamburg became more independent over time as compared to the settlers in the noncosmopolitan city of Braunschweig. Specifically, for each of the two independence measures employed (number of personal life tasks and preference for uniqueness) we estimated a GLM with settlement destination (Hamburg vs. Braunschweig) and length of stay (in years) in the new city as independent variables. For the number of personal (vs. relational and collective) life tasks we observed an interaction effect of settlement destination by length of stay, *F*_(1, 80)_ = 6.84, *p* = 0.01, indicating that the longer settlers lived in Hamburg the more personal life tasks they pursued (*r* = 0.33, *p* = 0.01). In contrast, there was no relation between length of stay and personal life tasks for settlers in Braunschweig (*r* = −0.14, *p* = 0.48). For the preference for uniqueness measure (liking of uncommon vs. common figures) however we did not observe a main or interaction effect with settlement destination or length of stay, *F*s < 1.92, *ps* > 0.17. These results suggest that both processes, self-selection and reinforcement of independence, may be involved in cosmopolitan settlement at least for some aspects of independence. Future studies may use longitudinal designs to gain greater insight into which aspects of independence are reinforced by settlement in cosmopolitan cities.

### Mediating mechanisms of cosmopolitan settlement

Concerning the mechanisms that mediate independent people's self-selection for cosmopolitan settlement, we hypothesized that cosmopolitan cities symbolize an independent lifestyle (e.g., freedom, self-realization, and nonconformity). Thus, independent people may feel attracted to settle in cosmopolitan cities via the mechanism of prototype matching between personality and location (Niedenthal et al., 1985). We should note that regardless of whether people's image of a city as cosmopolitan is accurate or inaccurate, in either case it may play an important role in guiding people's moving decisions (Phillips and Brunn, 1978; Fuguitt and Brown, 1990).

Future research may investigate whether other mechanisms, such as positive attitudes toward an independent life-style (Triandis, 1977), flexible social ties and weak social obligations in cosmopolitan cities (Yamagishi et al., 2012), or expected utility of settlement in cosmopolitan cities (DeJong and Fawcett, 1981) play a role in cosmopolitan settlement. Finally, the association of cosmopolitan cities with independence may fuel voluntary settlement by activating independent mind-sets. A recent series of studies supports this proposition by suggesting that participants primed with stimuli related to cosmopolitan cities were more independent on a number of cognitive, affective, and behavioral measures than those primed with noncosmopolitan cities (Sevincer et al., in preparation).

### Related approaches

#### Urban vs. rural mentality

Cosmopolitan cities tend to be urban although not all urban areas necessarily qualify as cosmopolitan. Investigating psychological differences between urban and rural residents is a longstanding research topic (Simmel, 1903). Studies suggest that residents in urban environments are generally more independent than those in more rural environments. Residents in urban (vs. rural) areas for instance regard collective aspects of their self as less important (Kashima et al., 2004), are more emotionally expressive (Matsumoto et al., 2009), and show a greater preference for uniqueness (Yamagishi et al., 2012). Our finding that more independent people prefer moving to cosmopolitan cities implies that the urban-rural difference in independence may in part be due to those settlers.

#### Residential mobility

It has been proposed that residential mobility (i.e., people's tendency to relocate) is associated with independence (Oishi and Kisling, 2009). Indeed, our finding in Study 2 that participants primed with independence (vs. interdependence) showed a greater willingness to relocate extends previous research on residential mobility by suggesting a causal link such that independence fosters residential mobility. At the same time, our research suggests that the destination of residential moves also needs to be taken into account. In other words, people's social orientation not only influences *whether* people move, but also *where* they move to.

#### Other person variables related to residential preferences

Finally, other person variables than social orientation (independence vs. interdependence) may influence residential preferences toward cosmopolitan cities. For example, because cosmopolitan cities are places of high diversity, people high in openness to experience may prefer moving there (Rentfrow et al., 2009). In addition, personality traits that were found to play a role in migration within nations may also be related to migration toward cosmopolitan cities. For instance, recent studies have found that low agreeableness and high extraversion were associated with migration within the U.S. (Jokela, 2009) and high sociability predicted migration from rural to urban municipalities in Finland over a 9-year period (Jokela et al., 2008).

#### Reasons for settlement

Traditional models of rural-urban migration emphasize the role of economic factors (the quantity and quality of available jobs; Harris and Todaro, 1970) for people's moving decisions. In addition, recent research conducted in Denmark suggests that another important reason why people move from the countryside to urban areas is to find a mate (Gautier et al., 2010). Future research on cosmopolitan settlement may further illuminate other reasons why people move to cosmopolitan cities by asking them their reasons for changing their place of residence.

## Conclusion

The present program of research illuminates the relationship between people's social orientation (independence vs. interdependence) and their likelihood to actively settle in cosmopolitan cities. In the 18 and 19th century the frontier in the Western U.S. symbolized unlimited opportunities, wealth, and freedom and, by attracting highly independent orientated people, shaped the nations' orientation toward independence. In the twenty-first century, the new frontiers may be cosmopolitan cities that value self-determination, personal goal pursuit, nonconformity, pluralism, tolerance, creativity, and openness to ideas, and may be an important factor in continuing shifts toward individualism in many cultures.

### Conflict of interest statement

The authors declare that the research was conducted in the absence of any commercial or financial relationships that could be construed as a potential conflict of interest.

## Footnotes

^1^Socioeconomic Status. Retrieved from: http://www.apa.org/topics/socioeconomic-status/index.aspx

^2^In Studies 1, 2, and 3 we performed all analyses a second time adding gender as a factor to explore possible interactions of our main predictors with gender. We did not observe any systematic interactions with gender. Moreover, in all studies the pattern of results did not change when gender was included in the analyses.

^3^Retrieved from: http://www.topuniversities.com/

^4^Due to time constraint we only assessed parent education as a proxy for SES in the sample from Braunschweig.
